# Enhancing Attention Network Spatiotemporal Dynamics for Motor Rehabilitation in Parkinson’s Disease

**DOI:** 10.34133/cbsystems.0293

**Published:** 2025-06-19

**Authors:** Guangying Pei, Mengxuan Hu, Jian Ouyang, Zhaohui Jin, Kexin Wang, Detao Meng, Yixuan Wang, Keke Chen, Li Wang, Li-Zhi Cao, Shintaro Funahashi, Tianyi Yan, Boyan Fang

**Affiliations:** ^1^ School of Medical Technology, Beijing Institute of Technology, Beijing, China.; ^2^ Parkinson Medical Center, Beijing Rehabilitation Hospital, Capital Medical University, Beijing, China.; ^3^ Advanced Research Institute of Multidisciplinary Science, Beijing Institute of Technology, Beijing, China.

## Abstract

Optimizing resource allocation for Parkinson’s disease (PD) motor rehabilitation necessitates identifying biomarkers of responsiveness and dynamic neuroplasticity signatures underlying efficacy. A cohort study of 52 early-stage PD patients undergoing 2-week multidisciplinary intensive rehabilitation therapy (MIRT) was conducted, which stratified participants into responders and nonresponders. A multimodal analysis of resting-state electroencephalography (EEG) microstates and functional magnetic resonance imaging (fMRI) coactivation patterns was performed to characterize MIRT-induced spatiotemporal network reorganization. Responders demonstrated clinically meaningful improvement in motor symptoms, exceeding the minimal clinically important difference threshold of 3.25 on the Unified PD Rating Scale part III, alongside significant reductions in bradykinesia and a significant enhancement in quality-of-life scores at the 3-month follow-up. Resting-state EEG in responders showed a significant attenuation in microstate C and a significant enhancement in microstate D occurrences, along with significantly increased transitions from microstate A/B to D, which significantly correlated with motor function, especially in bradykinesia gains. Concurrently, fMRI analyses identified a prolonged dwell time of the dorsal attention network coactivation/ventral attention network deactivation pattern, which was significantly inversely associated with microstate C occurrence and significantly linked to motor improvement. The identified brain spatiotemporal neural markers were validated using machine learning models to assess the efficacy of MIRT in motor rehabilitation for PD patients, achieving an average accuracy rate of 86%. These findings suggest that MIRT may facilitate a shift in neural networks from sensory processing to higher-order cognitive control, with the dynamic reallocation of attentional resources. This preliminary study validates the necessity of integrating cognitive–motor strategies for the motor rehabilitation of PD and identifies novel neural markers for assessing treatment efficacy.

## Introduction

Parkinson’s disease (PD) is a multisystem neurodegenerative condition characterized by the depletion of dopaminergic neurotransmitters in the basal ganglia, resulting in diminished synaptic connectivity and functional dysregulation within the cortico-subcortical network, which underlies both motor and nonmotor symptoms [1]. Motor rehabilitation has emerged as a novel adjunctive strategy in the management of PD, with multidisciplinary intensive rehabilitation therapy (MIRT)—a comprehensive intervention based on multimodal aerobic training and goal-oriented task design—demonstrating both short- and long-term benefits for motor and nonmotor symptoms in PD patients [2]. However, the impaired motor learning capacity in PD patients due to striatal dysfunction may significantly affect the efficiency of neuroplastic transformations during rehabilitation [3]. Randomized controlled trials have indicated that a minimum duration of 4 weeks of MIRT is required to elicit significant clinical functional improvements [4]. Notably, recent studies have shown that a 2-week MIRT intervention can effectively enhance overall motor function, gait coordination, balance control, and postural stability in PD patients [5]. Nevertheless, the neuroplastic mechanisms by which short-term MIRT induces motor improvement remain unclear, thereby constraining evidence-based decisions for optimizing individualized rehabilitation strategies and the precise allocation of clinical resources.

The core objectives of PD rehabilitation are to reconstruct habitual motor patterns, restore the precision of automated movements, and enhance the capacity for dynamic transitions between goal-directed behaviors and autonomous motor schemas. This process not only relies on plastic reconfiguration of the cortico-basal ganglia circuitry but also crucially involves functional compensation through the activation of attentional networks, including the parietal association cortex [6]. The therapeutic mechanism of motor rehabilitation may entail the reallocation of attentional resources to counteract deficits in motor automation, thereby reinforcing cortico-subcortical pathways and activating compensatory networks such as the frontoparietal network (FPN) [7]. The engagement of higher-order cognitive control is pivotal for the early stages of motor learning and execution control [8]. The observed motor functional gains in PD patients following systematic rehabilitation training are primarily attributed to the enhancement of the attention and sensorimotor compensation circuits [9]. High-intensity, goal-based aerobic and challenging practice, such as MIRT, effectively induces dynamic reprogramming of dopaminergic signaling pathways and the reallocation of attentional resources, underpinning the neuroplastic basis for improved motor function [10]. Neuroimaging evidence further indicates that the topological characteristics of higher-order associative networks related to sensorimotor control and execution in PD patients change significantly, such as the functional gradient of the dorsal attention network (DAN) and the structural gradient of the default mode network (DMN), which are correlated with their motor function scores [11]. The attentional mechanism not only initiates goal-directed behaviors but also plays a central role in the functional reorganization of motor circuits in PD patients [12]. Notably, the severity of PD symptoms is mainly determined by the efficacy of the premotor–parietal cortical compensation network rather than by the degree of basal ganglia dysfunction [13]. Short-term motor rehabilitation may prioritize the induction and maintenance of cortical compensation mechanisms over the physiological restoration of basal ganglia function in PD [14].

The characterization of spatiotemporal brain dynamics following PD treatment has primarily focused on identifying altered patterns in time-varying functional connectivity (FC) [15], with FC temporal attributes strongly linked to motor deficit severity [8]. Restored parietal–thalamic connectivity and strengthened frontal–parietal networks may enhance inferior parietal neurovascular coupling in PD, thus remodeling executive attention functions during early-phase motor learning [16]. Pharmacological treatments for PD, such as L-dopa, elicit concurrent increases in connectivity within the motor network, DAN, and DMN, as demonstrated by simultaneous electroencephalography (EEG)–functional magnetic resonance imaging (fMRI) data acquisition [17]. However, the precise spatiotemporal reconfiguration resulting from motor rehabilitation as indicated by EEG dynamics and fMRI network transitions remains elusive [18]. To bridge this gap, coactivation patterns (CAPs) of fMRI [19] and EEG microstate analysis [20] represent data-driven advanced techniques that quantify whole-brain network dynamics at sub-second temporal resolution and map transient spatiotemporal patterns of sensory, attentional, and default mode networks. EEG microstates sensitively characterize the clinical phenotypic heterogeneity, therapeutic response variability, and prognostic trajectories in PD [21], while fMRI CAPs shed light on the dynamic network reorganization associated with symptom manifestation [22]. EEG microstate dynamics in PD patients treated with subthalamic nucleus deep brain stimulation exhibit unique patterns that hold potential as biomarkers for both motor and nonmotor symptomatology [23]. EEG microstate analysis elucidates the therapeutic impact of repetitive transcranial magnetic stimulation in PD patients, with a notable increase in microstate D activity being significantly associated with enhanced motor cognitive function [24]. The CAP pattern characterized by visual network deactivation and thalamic activation was increased in amplitude after transcranial alternating current stimulation treatment in PD patients [25].

By integrating EEG microstate analysis with fMRI CAPs, our study aims to uncover the underlying mechanisms of PD motor rehabilitation after a 2-week MIRT with greater precision. This integrated approach not only validates prior electrophysiological and imaging findings but also identifies new targets for neuroplasticity that are relevant to motor rehabilitation in PD. We hypothesize that 2-week MIRT enhances motor behavior by activating frontoparietal and attentional networks through a cortical compensation mechanism, with EEG microstate temporal precision complementing the fMRI CAPs’ spatial specificity.

## Materials and Methods

### Participants and study design

**Fig. 1.**
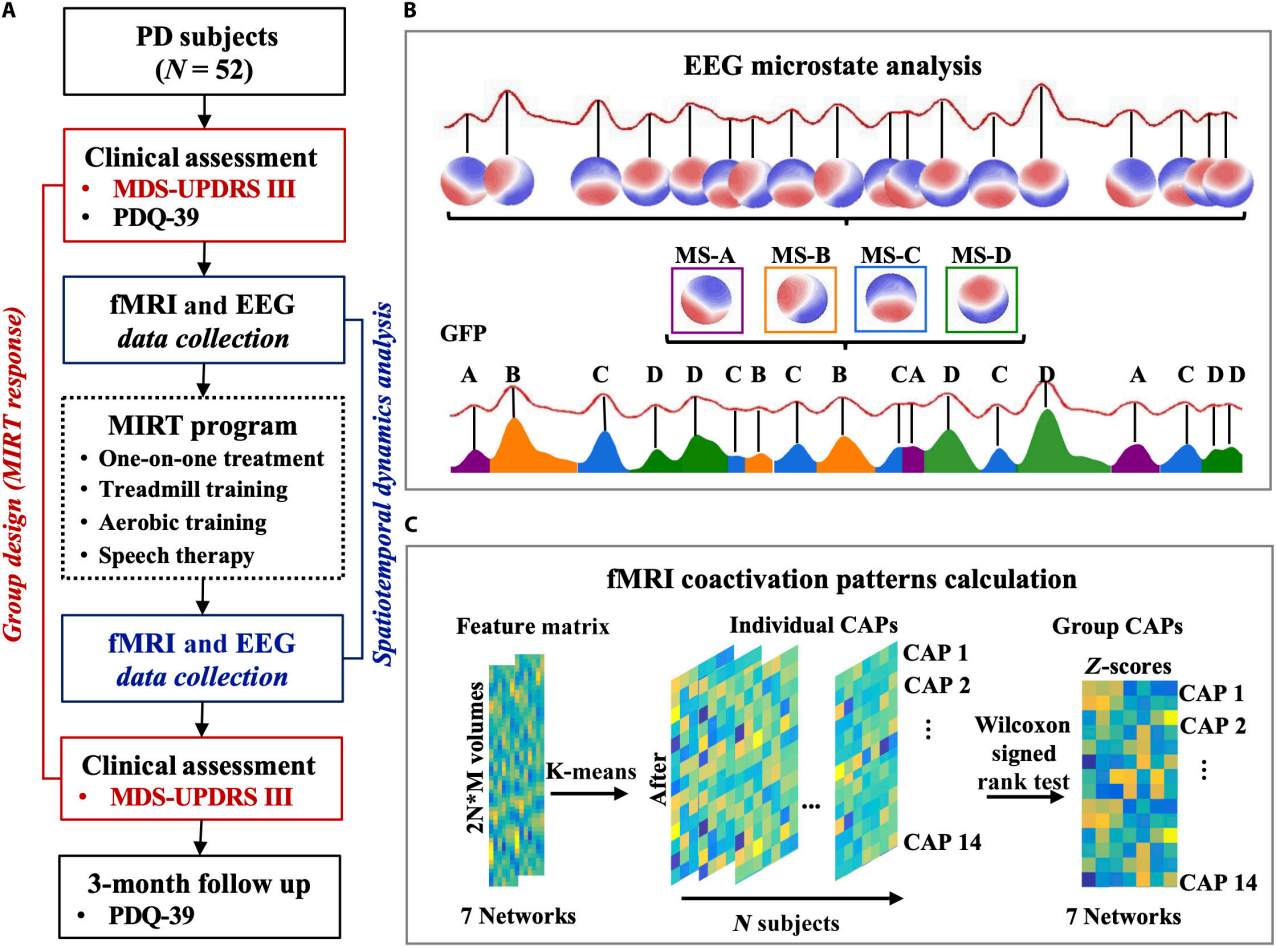
Workflow of analysis. (A) The pipeline of the MIRT treatment and data collection. (B) The pipeline of the EEG microstate analysis. (C) The pipeline of the fMRI coactivation pattern calculation. EEG, electroencephalography; GFP, global field power; fMRI, functional magnetic resonance imaging; CAPs, coactivation patterns; MIRT, multidisciplinary intensive rehabilitation therapy; MDS-UPDRS III, Movement Disorders Society-Sponsored Revision of the Unified PD Rating Scale part III; PDQ-39, 39-item PD questionnaire.

Eighty-one PD patients were recruited into the cohort from April to November 2020. Fifty-two patients with complete evaluation materials were selected for the Multidisciplinary Rehabilitation Registration Study on PD (registered in the Chinese Clinical Trial Registry under the identifier 2000033768). The inclusion and exclusion criteria are detailed in the Supplementary Materials. All participants underwent a 2-week MIRT program and underwent clinical assessments and fMRI and EEG data collection before and after the intervention. Participants were categorized into responders and nonresponders based on a 3.25-point reduction in Movement Disorders Society-Sponsored Revision of the Unified PD Rating Scale part III (MDS-UPDRS III) scores [26], which is the threshold for minimal clinically important difference (Fig. 1A).

### MIRT program

The MIRT program was administered in a hospital environment, comprising 4 daily sessions of 30 to 60 min each, 5 days a week for 2 weeks. The program included personalized physical therapy, treadmill and aerobic training, and speech therapy. Patients maintained their standard medical regimen, which remained consistent throughout the study. Participants provided written informed consent in accordance with the Helsinki Declaration, and the study was ethically reviewed and approved by the Ethics Committee of Beijing Rehabilitation Hospital, Capital Medical University (2020bkky010). The specific MIRT process is outlined in the Supplementary Methods.

### Clinical assessments

The primary short-term outcome of the MIRT program was assessed within 1 to 2 days. MDS-UPDRS III was used to measure changes in motor symptoms, including rigidity, tremor, axial, and bradykinesia. Long-term effects were evaluated after 3 months using the 39-item PD Questionnaire (PDQ-39). During MIRT and follow-up, patients received stable medication without adjustments. Assessments were conducted in the patients’ “on” state (1 to 2 h after medication) by the same physical therapist and neurologist.

### EEG data acquisition and preprocessing

EEG data from 52 PD patients were collected using a 64-channel system (ANT Neuro GmbH) with impedance below 5 kΩ and a 1,000 Hz sampling rate. The Cz channel served as the acquisition reference. Three minutes of closed-eye EEG data were captured pre- and post-MIRT. EEG data were preprocessed using the EEGLAB toolbox in MATLAB (Version 2017a, The MathWorks, Natick, MA, USA). An independent component analysis was used to remove artifacts from the data within all channels. The artifact-free EEG data were down-sampled to 500 Hz and bandpass filtered using the basic finite impulse response filter to include a broadband frequency range of 1 to 30 Hz.

### Dynamic brain states and metrics by EEG microstate analysis

The artifact-free resting-state EEG data were analyzed using CARTOOL software to extract microstates, with global field power determined for each topography. The topographic maps of maximum global field power values were created, and the data were clustered into 4 microstate classes (A, B, C, and D) using modified *k*-means spatial cluster analysis [27]. A comprehensive systematic parameter optimization was carried out to enhance the efficiency and accuracy of the clustering process [28]. The *k*-means algorithm was initialized randomly 50 times to mitigate the risk of converging to a local optimum. Through the minimization of the cross-validation error, the optimal number of clusters was determined to be 4. Group model maps were computed from individual model maps before and after MIRT for both response and nonresponse groups. These model maps were back-fitted to the original data for each participant (Fig. 1B). The back-fitting process involved aligning the individual microstate sequences with the group model maps to ensure consistency in microstate classification across participants. Additionally, to enhance the reliability of the transition metrics, a smoothing technique was applied to the state sequences, and transitions with durations less than 30 ms, which were considered physiologically implausible transient switches, were excluded. Microstate parameters such as global explained variance, duration, occurrence, coverage, and transition percentage were calculated for the sorted microstates to characterize their spatiotemporal dynamics. The global explained variance reflects the contribution of each microstate template to the global variance of the EEG signal. Temporal coverage quantifies the relative persistence of a microstate by measuring its cumulative presence as a percentage of total recording time, while duration reflects the average time span of individual microstate episodes, indicating temporal stability. Occurrence captures the activation frequency of a microstate per unit time, revealing its propensity for recurrent engagement. Finally, transition probability delineates the directional shifts between microstates, defined as the conditional likelihood of transitioning from one microstate to another within a single time step, thereby mapping the temporal hierarchy of brain network reconfigurations [29].

### MRI data acquisition and preprocessing

Resting-state functional and structural MRI data from 51 out of 52 enrolled patients with PD were successfully recorded using a 3.0-T MRI scanner (Pioneer, GE, USA); one subject’s data were corrupted during data collection. Blood oxygen level-dependent (BOLD) functional images were collected using a gradient-recalled echo-planar imaging sequence (echo time = 35 ms, repetition time = 2,000 ms, flip angle = 90°, interslice gap = 0 mm, field of view = 280 × 280 mm^2^, matrix = 128 × 128, slice thickness = 4.0 mm, and slice number = 40). The resting-state functional MRI data were preprocessed by using SPM 12 (https://www.fil.ion.ucl.ac.uk/spm/) and the toolbox DPABI 4.4 in MATLAB 2014a. For the details of the MRI data preprocessing, see Supplementary Methods.

### Dynamic brain states and metrics by CAP analysis

This study employed a sliding-window approach implemented in the GRETNA toolbox (MATLAB R2014a) to characterize dynamic brain activity [30]. Regional time series were generated by spatially averaging BOLD signals across all voxels within each region of interest (ROI). Temporal correlations (Pearson correlation coefficients) between ROIs were computed through sliding-window analysis to quantify time-resolved fluctuations in FC. The Yeo-7 network segmentation scheme effectively captures the brain’s core functional systems and demonstrates superior sensitivity to dynamic neural activity, with established reliability and neurobiological interpretability [31]. Given our observation that EEG microstate parameters predominantly exhibited significant alterations in microstates C and D, which may be associated with higher-order cognitive control networks such as the DMN and DAN, we adopted this parcellation framework to systematically characterize intersubject functional variations and align with prior neuroimaging findings [11]. The brain was segmented into 7 networks using a 1,000-brain FC MRI atlas, namely, the visual network (VN), somatomotor network (SMN), DAN, ventral attention network (VAN), limbic network (LN), FPN, and DMN. The *k*-means clustering identified brain activation patterns or states, with cosine similarity used as the cluster distance measure. Clustering was repeated 1,000 times with different random initializations, and the solution with the best data separation was chosen. The *k* value was set to 14 to ensure high similarity within and high dissimilarity between clusters (Fig. S1). For each participant, *k* individual CAPs were derived from the averaged volumes. Wilcoxon signed-rank tests evaluated the median value of ROI-wise signals and compared *z*-scores for each CAP to identify significant coactivation and inactivation patterns (Fig. 1C). Fractional occupancy, dwell time, and appearance rate were calculated separately for each subject and each state of CAP.

### Clinical effect prediction

To evaluate the predictive efficacy of MDS-UPDRS III, EEG microstate dynamics, and fMRI CAPs for MIRT outcomes in PD, a binary support vector machine (SVM) classifier was implemented in MATLAB. The model incorporated Bayesian optimization to automatically determine optimal kernel parameters, with feature selection focusing on MDS-UPDRS III scores and neurophysiological metrics demonstrating clinical relevance: the occurrences of EEG microstates C and D, along with the dwell time of the fMRI-derived CAP (DAN+ VAN−). Pre-MIRT data from PD cohorts (*n* = 51, after excluding one unpaired sample from the original 52 participants to ensure modality alignment) were used in the analysis. A 5-fold cross-validation framework with nested design was applied during training to prevent data leakage. To mitigate stochastic variability, 5 independent experimental iterations were conducted with randomized data splits. Model performance was quantified using the decision values derived from the SVM hyperplane, with classification thresholds optimized to maximize sensitivity and specificity. Final results were reported as the mean ± standard error (SE) of accuracy, sensitivity, and specificity across all iterations, ensuring statistically robust evaluation of the neurodynamic predictors.

### Statistical analysis

The normality of demographic, clinical, and neurophysiological variables was assessed using the Shapiro–Wilk test. For data that followed a normal distribution, paired *t* tests (for within-group comparisons) or independent *t* tests (for between-group comparisons) were conducted. For data that violated the assumption of normality, nonparametric tests were employed, including the Wilcoxon signed-rank test (for within-group comparisons) and the Mann–Whitney *U* test (for between-group comparisons). To examine neural variables, a repeated-measures analysis of variance was performed, with group (responders vs. nonresponders) as the between-subjects factor and time point (pre- vs. post-MIRT) as the within-subjects factor. This analysis focused on changes in neurophysiological metrics, including microstate parameters, transition probabilities, and CAP parameters. The relationships between the rate of reduction in MDS-UPDRS III scores, the rate of change in microstate parameters, and temporal CAP metrics were evaluated using Spearman’s rank correlation coefficient. A 95% confidence interval was applied for all analyses, and the significance threshold was set at *α* = 0.05. To account for multiple comparisons, the Bonferroni correction was applied, with adjusted *P* values < 0.05 considered statistically significant.

## Results

### Clinical outcomes

Among 52 participants, 24 PD patients (46.2%) demonstrated significant motor improvement following MIRT, with MDS-UPDRS III scores decreasing by >3.25 points (*t* = 10.551, *P*_BON_ < 0.001, 95% confidence interval [CI] [5.326, 7.924]). Responders exhibited significant reductions in bradykinesia (*t* = 6.151, *P*_BON_ < 0.001, 95% CI [2.959, 5.958]) and a significant improvement in quality of life, as evidenced by decreased PDQ-39 scores at the 3-month follow-up (*z* = −3.744, *P*_BON_ < 0.001, 95% CI [0, 0.117]). In contrast, nonresponders showed no significant changes in MDS-UPDRS III (including subscales) or PDQ-39 scores (all *P* > 0.05). Baseline clinical and demographic characteristics did not differ significantly between responders and nonresponders (all *P* > 0.05; Table 1).

**Table 1.** Demographics and clinical data in both groups before and after MIRT

Characteristic	Responding group (*n* = 24)			Nonresponding group (*n* = 28)			Baseline
	Before mean (SD)	After mean (SD)	*P*_BON_ value	Before mean (SD)	After mean (SD)	*P* value	*P* value
Sex (F/M)	11/13			11/17			0.634^a^
Age (years)	62.79 (6.81)			61.18 (6.73)			0.396^b^
Education (years)	11.73 (4.37)			11.36 (4.53)			0.803^c^
Disease duration (months)	86.13 (53.46)			76.03 (41.07)			0.376^c^
L-dopa equivalent dose (mg)	611.77 (348.04)			529.05 (293.21)			0.569^c^
Hoehn–Yahr stage	2.35 (0.52)			2.32 (0.41)			0.857^c^
MDS-UPDRS III score	40.42 (11.02)	33.79 (10.93)	<0.001^d^	36.25 (10.76)	37.11 (11.90)	0.217^d^	0.175^c^
Tremor subscale score	4.21 (3.93)	3.54 (3.50)	0.106^e^	3.50 (3.67)	4.00 (4.39)	0.100^d^	0.586^c^
Bradykinesia subscale score	19.92 (7.23)	15.46 (6.65)	<0.001^d^	18.71 (6.14)	18.93 (6.46)	0.689^d^	0.550^c^
Rigidly subscale score	9.75 (2.19)	9.33 (1.97)	0.904^d^	8.75 (1.82)	9.14 (2.09)	0.163^d^	0.078^c^
Axial subscale score	6.54 (3.68)	5.46 (2.90)	0.080^d^	5.29 (2.57)	5.04 (2.89)	0.199^d^	0.171^c^
PDQ-39 score	23.60 (9.92)	15.17 (9.29)	<0.001^e^	24.19 (8.85)	20.49 (10.15)	0.067^d^	0.575^c^

MIRT, multidisciplinary intensive rehabilitation therapy; SD, standard deviation; F, female; M, male; MDS-UPDRS III, Movement Disorders Society-Sponsored Revised Unified Parkinson’s Disease Rating Scale part III; PDQ-39, 39-item Parkinson’s Disease Questionnaire

^a^Chi-squared test.

^b^Independent-samples test.

^c^Mann–Whitney *U* test.

^d^Paired-sample test.

^e^Wilcoxon signed-ranks test.

### Effects of MIRT on EEG microstate dynamics

**Fig. 2.**
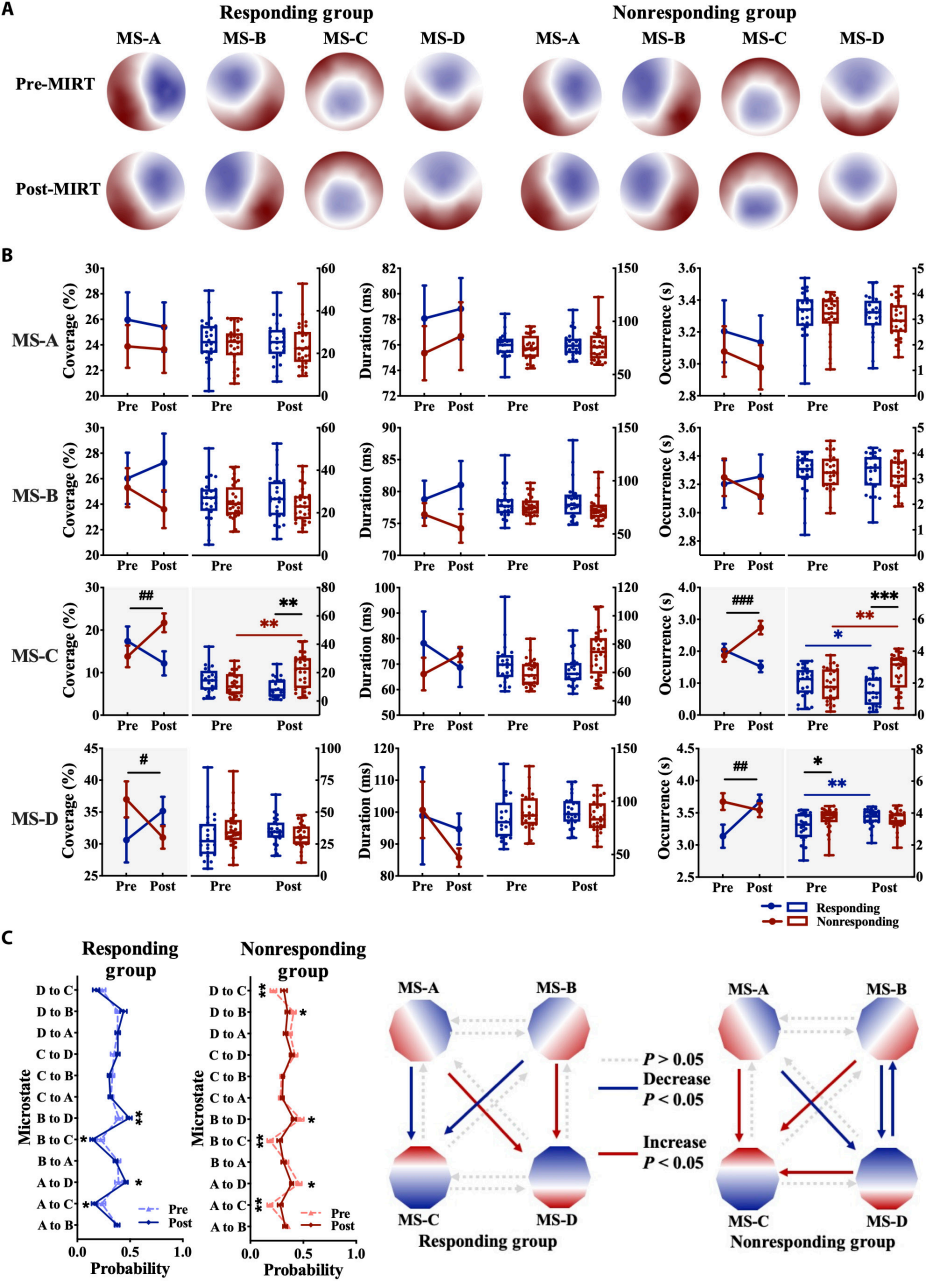
Spatial configurations and temporal dynamics of EEG microstates (classes A to D). (A) EEG topography plots showing spatial configurations of microstates (classes A to D) pre- and post-MIRT. Colors represent the global field power polarity. (B) Temporal parameters (time coverage, occurrence, and duration) are presented as line plots with box plots (mean, range, and individual data). #*P* < 0.05; ##*P* < 0.01; ###*P* < 0.001 (interaction effect); *P < 0.05; **P < 0.01; ***P < 0.001 (black: intra-group; red: nonresponders; blue: responders), all corrected. (C) Line plots show the EEG microstate transition probabilities and transition patterns between classes. *P < 0.05 and **P < 0.01 (all corrected). MS-A, -B, -C, and -D, microstate class A, B, C, and D.

Fig. 2A displays the group-averaged microstate topographies before and after MIRT. Microstates A and B exhibited diagonal orientations, microstate C showed an anterior–posterior axis, and microstate D was centered in the frontal lobe. There was no significant interaction effect of MIRT × group for the global explained variance (*F* = 0.998, *P* > 0.05, $\eta_{p}^{2}$ = 0.059). Significant MIRT × group interactions were observed for microstate C coverage (*F* = 12.137, *P* = 0.001, $\eta_{p}^{2}$ = 0.195), with nonresponders showing increased coverage post-MIRT compared to baseline (*P*_BON_ = 0.003) and exceeding responders’ levels (*P*_BON_ = 0.009). Microstate C occurrence also showed significant interactions (MIRT × group) (*F* = 16.135, *P* < 0.001, $\eta_{p}^{2}$ = 0.244), decreasing in responders (*P*_BON_ = 0.048) but increasing in nonresponders (*P*_BON_ = 0.001) post-MIRT, with significant intergroup differences (*P*_BON_ < 0.001). Microstate D occurrence demonstrated similar interactions (*F* = 9.279, *P* = 0.004, $\eta_{p}^{2}$ = 0.157), increasing only in responders (*P*_BON_ = 0.002) post-MIRT, who had lower baseline levels than nonresponders (*P*_BON_ = 0.017) pre-MIRT. While microstate D coverage showed significant interactions (*F* = 5.130, *P* = 0.028, $\eta_{p}^{2}$ = 0.093), simple effects were nonsignificant (all *P*_BON_ > 0.05). Microstates A and B remained unaffected by MIRT or group (all *P* > 0.05) (Fig. 2B). Transition probabilities revealed significant MIRT × group interactions for the following: (a) microstate A/B/D→C transitions (A→C: *F* = 16.704, *P* < 0.001, $\eta_{p}^{2}$ = 0.250; B→C: *F* = 16.151, *P* < 0.001, $\eta_{p}^{2}$ = 0.244; D→C: *F* = 11.353, *P* = 0.001, $\eta_{p}^{2}$ = 0.185), (b) microstate A/B→D transitions (A→D: *F* = 9.106, *P* = 0.004, $\eta_{p}^{2}$ = 0.154; B→D: *F* = 13.895, *P* < 0.001, $\eta_{p}^{2}$ = 0.217), and (c) microstate D→B transitions (*F* = 8.750, *P* = 0.005, $\eta_{p}^{2}$ = 0.149). Responders showed significantly decreased microstate A/B→C transitions (A→C: *P*_BON_ = 0.023; B→C: *P*_BON_ = 0.016) but significantly increased microstate A/B→D transitions (A→D: *P*_BON_ = 0.043; B→D: *P*_BON_ = 0.004). Conversely, nonresponders exhibited significantly increased microstate A/B/D→C transitions (A→C: *P*_BON_ = 0.001; B→C: *P*_BON_ = 0.002; D→C: *P*_BON_ = 0.003), along with elevated microstate A/B→D and microstate D→B transitions (A→D: *P*_BON_ = 0.033; B→D: *P*_BON_ = 0.032; D→B: *P*_BON_ = 0.030) (Fig. 2C).

### Effects of MIRT on CAP dynamics

**Fig. 3.**
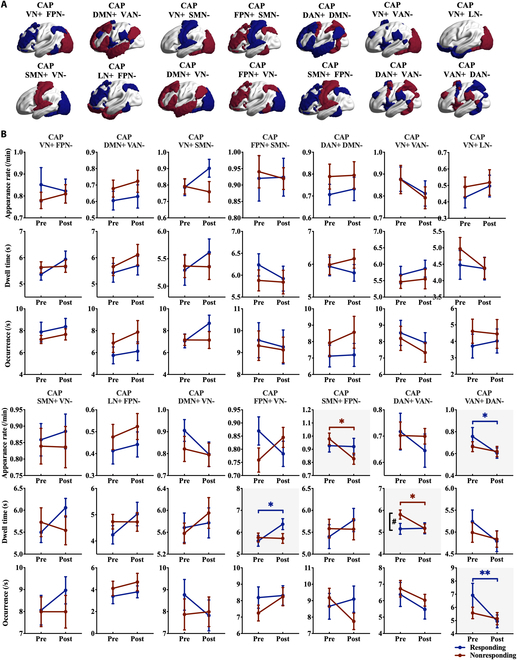
Fourteen brain activity states of coactivation patterns and the temporal metrics. (A) Fourteen coactivation patterns (CAPs) plots show the activated (red, highest *z*-score) and deactivated (blue, lowest *z*-score) networks. VN, visual network; SMN, somatomotor network; DAN, dorsal attention network; VAN, ventral attention network; LN, limbic network; FPN, frontoparietal network; DMN, default-mode network. (B) Line charts display average data and standard error of the CAP temporal metrics for the appearance rates, dwell time, and fractional occupancy pre- and post-MIRT. *P < 0.05 and **P < 0.01, intra-group (red: nonresponders; blue: responders), all corrected; #*P* < 0.05, intergroup (all corrected).

Fourteen functional CAPs were identified based on network-specific peak activation and deactivation patterns (Fig. 3A). CAP *z*-scores were computed using Wilcoxon signed-rank tests (Fig. S2). There was a significant MIRT × group interaction specifically for the dwell time of CAP (FPN+ VN−) (*F* = 4.155, *P* = 0.047, $\eta_{p}^{2}$ = 0.078). Responders showed a significant increase in post-MIRT (*P*_BON_ = 0.012), while nonresponders showed no significant difference (*P*_BON_ > 0.05). No significant interactions were observed for other CAP temporal parameters (all *P* > 0.05). Notably, responders exhibited significantly decreased CAP (VAN+ DAN−) appearance rate (*P*_BON_ = 0.025) and occurrence (*P*_BON_ = 0.004) following MIRT. In contrast, nonresponders demonstrated reduced dwell time in the adversarial network CAP (DAN+ VAN−) (*P*_BON_ = 0.030) and decreased CAP (SMN+ FPN−) appearance rates (*P*_BON_ = 0.037) (Fig. 3B).

### Clinical correlations of spatiotemporal dynamics indicators

**Fig. 4.**
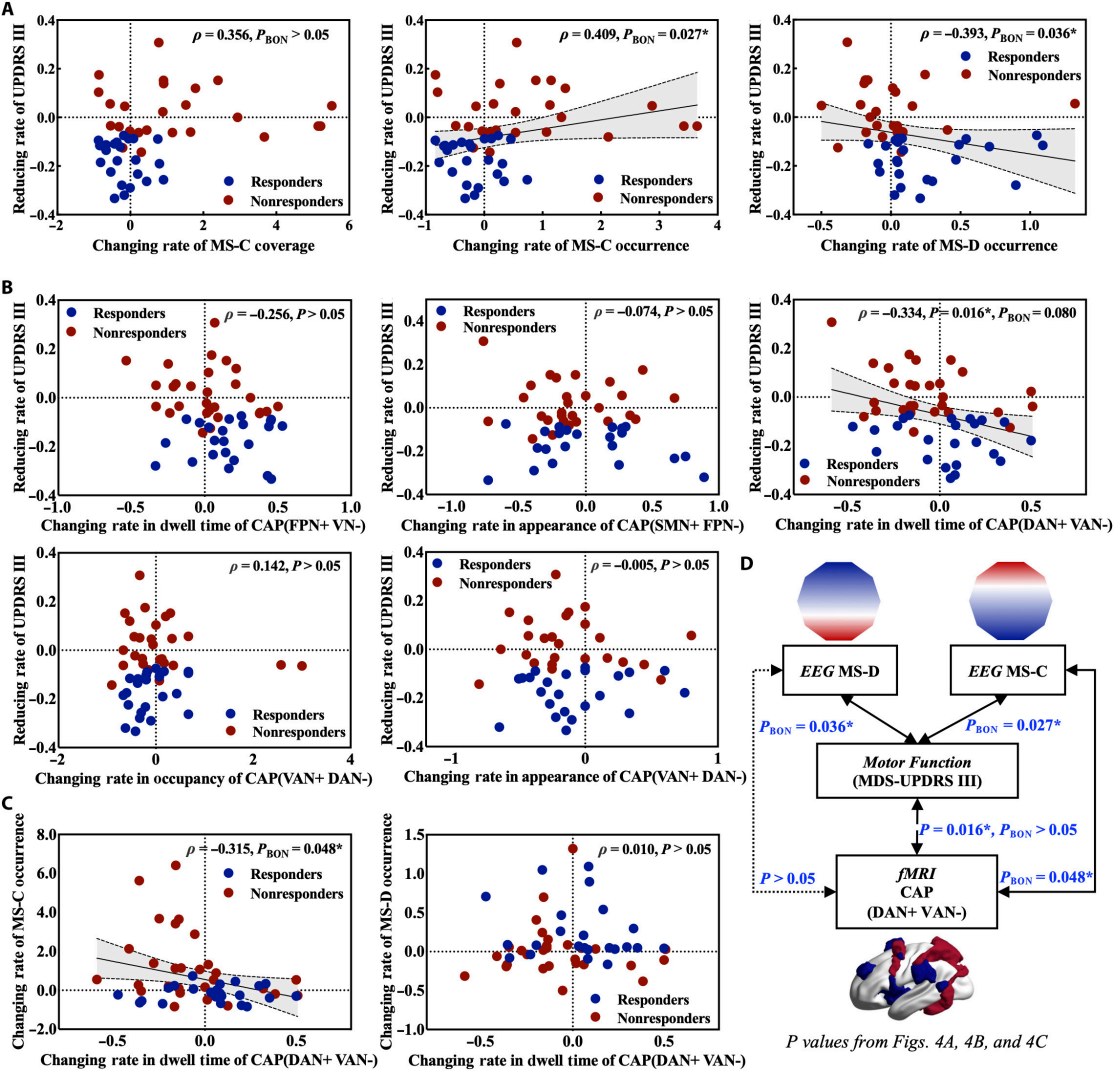
Spatiotemporal dynamic indicators clinical correlations. (A) Scatter plots display the clinical correlation of EEG microstate temporal parameters with significant differences. *Corrected *P* < 0.05. (B) Scatter plots display the clinical correlation of fMRI CAP temporal parameters with significant differences. *Corrected *P* < 0.05. (C) Scatter plots display the EEG-fMRI temporal parameter correlations. *Corrected *P* < 0.05. (D) Schematic overview of clinical correlations between fMRI, EEG temporal dynamics, and motor function. TP, transition probability; MS-A, -B, -C, and -D, microstate class A, B, C, and D; CAP, coactivation pattern.

Fig. 4A illustrates that among MIRT-modulated EEG microstate temporal parameters, only the change rates of microstate C and D occurrence showed significant correlations with MDS-UPDRS III reduction rate (microstate C: *ρ* = 0.409, *P*_BON_ = 0.027; microstate D: *ρ* = −0.393, *P*_BON_ = 0.036). Specifically, there is a significant negative correlation between the change rate of microstate D occurrence and the reduction rate of Bradykinesia subscale scores (*ρ* = −0.394, *P*_BON_ = 0.012) (Fig. S3A). Additionally, the change rates from microstate A/B → D transitions correlated significantly with MDS-UPDRS III (Fig. S3B) and Bradykinesia subscale score reduction rates (Fig. S3C). Fig. 4B reveals that among fMRI CAP temporal parameters modulated by MIRT, only CAP (DAN+ VAN−) dwell time change rate showed a significant negative correlation with MDS-UPDRS III reduction rate (*ρ* = −0.334, *P* = 0.016, *P*_BON_ = 0.080). The fMRI CAP temporal parameters showed no significant association with bradykinesia symptom improvement specifically (*P* > 0.05) (Fig. S4). Furthermore, Fig. 4C illustrates that CAP (DAN+ VAN−) dwell time change rate negatively correlated with microstate C occurrence (*ρ* = −0.315, *P*_BON_ = 0.048) but showed no significant association with microstate D occurrence (*P*_BON_ > 0.05). Fig. 4D demonstrates significant correlations between spatiotemporal brain metrics and clinical scale scores and provides an integrative synthesis of the key findings presented in Fig. 4A to C. Furthermore, the spatiotemporal dynamics of EEG (microstate C and D occurrences) and fMRI CAP (DAN+ VAN− dwell time) demonstrated significant predictive value for MIRT outcomes in PD. Table 2 presents the predictive performance of an SVM classifier in distinguishing MIRT responders from nonresponders. The mean classification accuracy across 5 iterations of 5-fold cross-validation demonstrated the following hierarchy: multimodal signal (highest accuracy) > EEG microstate > fMRI CAP > MDS-UPDRS III (lowest accuracy).

**Table 2.** Evaluation of the SVM-based MIRT response prediction model by 5 times 5-fold cross validation (mean ± SE)

Index	Accuracy (%)	Sensitivity (%)	Specificity (%)
MDS-UPDRS III	50.67 ± 1.63	48.57 ± 3.50	52.50 ± 2.50
fMRI CAP	76.00 ± 1.63	72.38 ± 3.50	78.61 ± 2.31
EEG microstate	82.66 ± 3.23	78.45 ± 2.45	86.00 ± 5.79
EEG microstate+ fMRI CAP	86.00 ± 2.67	80.47 ± 2.28	89.72 ± 4.47

SVM, support vector machine; MIRT, multidisciplinary intensive rehabilitation therapy; SE, standard error; MDS-UPDRS III, Movement Disorders Society-Sponsored Revised Unified Parkinson’s Disease Rating Scale part III; fMRI, functional magnetic resonance imaging; CAP, coactivation pattern; EEG, electroencephalography

## Discussion

This study primarily elucidates the spatiotemporal dynamics of brain networks in PD patients following MIRT. A case–control design involving 52 early-stage PD patients revealed that 24 responders exhibited clinically meaningful improvements in motor performance—particularly bradykinesia—after a 2-week MIRT intervention, suggesting that individuals with PD retain sufficient motor learning capacity to induce neuroplastic adaptations. Resting-state EEG analyses demonstrated that responders showed a decreased occurrence of microstate C and an increased occurrence of microstate D. Moreover, resting-state fMRI revealed prolonged coactivation of the DAN and deactivation of the VAN, which correlated significantly with motor symptom improvement. These findings indicate that MIRT may drive dynamic network reorganization, characterized by a shift from predominant sensory processing toward enhanced engagement of higher-order cognitive control systems. This neurofunctional reconfiguration likely facilitates the reallocation of attentional resources to optimize motor learning efficacy.

The functional reorganization of attentional networks may be closely associated with the pathogenesis of gait disorders in PD [32]. Microstate D is considered to reflect the activity of the frontoparietal attention network, which is closely linked to visual attention and is a critical component of the motor learning circuit [33]. Notably, EEG findings revealed that the increase in microstate D occurrence following MIRT was significantly correlated with improved motor function exclusively in the responder group, with no significant changes observed in the nonresponder group. Further analysis revealed a distinct divergence in microstate transition patterns. Responders exhibited significantly increased post-MIRT transition probability from microstate B to D, whereas nonresponders displayed the opposite trend, which may suggest that this discrepancy stems from compensatory visual network abnormalities in early-stage PD pathogenesis. Evidenced by the elevated temporal and spatial variabilities of the EEG microstate B network in PD patients compared to healthy subjects [34], previous studies have shown that there is visual network dysfunction in PD patients with freezing of gait [35]. In conjunction with our fMRI CAP results, responders exhibited an increase in the duration of FPN coactivation and VN deactivation following MIRT. The FPN, connected to the DAN, mediated top-down perceptual attention regulation [36]. This observation aligns with previous research indicating that frontal network connectivity modulation during treadmill training can elicit the recruitment of attentional networks in patients with PD [37]. Furthermore, the progressive degeneration of dopaminergic neurons in the substantia nigra is a core pathological substrate for the characteristic motor symptoms of PD, such as tremor and bradykinesia [38]. Levodopa treatment has been shown to ameliorate bradykinesia in patients with PD by modulating microstate D [39]. Our study revealed a significant association between the augmentation of microstate D and the alleviation of bradykinesia in patients with PD. Microstate D, as a marker sensitive to dopaminergic regulation, has been reported in attention deficit hyperactivity disorder [40] and schizophrenia [41], both of which feature abnormal dopamine transmission. The significant improvement in bradykinesia among responders in this study may stem from the enhancement of attentional network function induced by early synaptic plasticity, a mechanism consistent with the up-regulation of dopamine D2 receptor density following intensive treadmill training in early-stage patients with PD [42]. The prefrontal cortex and the top-down frontal attention control network play a significant role in the walking function of patients with PD [43]. DAN and VAN are primarily responsible for exogenous attentional reorientation and endogenous attention orientation, respectively [44]. Patients with PD experience a diminished ability to counteract bottom-up interference due to basal ganglia damage, affecting top-down attentional control [45]. The results of our clinical correlation analysis indicate that the prolongation of DAN coactivation/VAN deactivation pattern duration of fMRI is significantly associated with the improvement of motor symptoms, with responders showing a decrease in the appearance rate of VAN coactivation/DAN deactivation patterns in contrast to the nonresponder group. The mean dwell time of positive coupling between the SMN and the FPN in PD patients is compromised, potentially resulting in the motor cortex’s diminished capacity to process rapidly adaptive top-down control information promptly and accurately [8]. Within the CAP (DAN+ VAN−), which is intricately associated with the efficacy of MIRT in motor rehabilitation, the FPN (*z*-score: −1.016) and the SMN (*z*-score: 1.076) manifested a pattern of negative activation (Fig. S2). This reconfiguration of network activation is likely to bolster top-down control processes and mitigate motor symptoms. Therefore, the enhancement of DAN function may facilitate the alleviation of motor symptoms by the top-down control network in patients with PD.

Additionally, a significant opposite trend in microstate C was observed between the responder and nonresponder groups. Microstate C exhibits a robust association between its global field power and degree centrality within the DMN, which aligns with the network’s core functional hubs [46]. Mechanistically, its dynamic instability correlates with impaired attentional shifting efficiency, while task-induced suppression of its temporal parameters (e.g., duration, occurrence) mirrors the task-negative modulation of DMN [47]. Recent studies suggest that overconnectivity between the DMN and the visual network may contribute to gait freezing in PD [48]. Clinical correlation analysis revealed that a decrease in the occurrence of microstate C was significantly positively associated with the improvement of motor symptoms. This was indicated by the decrease in the temporal parameter of microstate C in responders and the increases in this parameter in nonresponders, following MIRT, suggesting that DMN suppression may facilitate motor recovery by enhancing executive functions and the mobilization of attentional resources. Moreover, the transition probabilities from microstate B to C in the responder group were significantly decreased after MIRT. PD patients are associated with failure of top-down processing of attentional networks, aberrant coupling of the DMN with VN, and disconnection between the thalamus and posterior brain areas, leading to aberrant disinhibition of the DMN [49]. Studies on aerobic training within MIRT have confirmed that increased prefrontal activity plays a crucial role in improving executive functions and motor behavior in patients with PD [50]. Further analysis indicated a significant negative correlation between the incidence of microstate C and the duration of DAN coactivation/VAN deactivation patterns. Given that the DAN is closely connected to the sensorimotor cortex and plays a central role in visuospatial attention [51], DMN activity, with its deactivation typically synchronized with goal-directed DAN activation, is subject to suppression by top-down attentional control [52]. In patients with PD, impaired deactivation of the DMN may be a direct consequence of dopaminergic depletion, contributing to deficits in executive control [53]. The reduction in microstate C accompanied by an increase in microstate D among responders may reflect a dynamic reallocation of attentional resources from internal mentation (DMN) to external task execution (DAN), thereby optimizing the efficiency of motor learning. Moreover, the increased transition probabilities from microstate B to D and A to D in the responder group were significantly correlated with motor improvement. Considering the topological characteristics of microstates B and A, which closely correspond to the visual and auditory networks, respectively [54], this dynamic reorganization from sensory to attentional networks may signify a neural mechanism for enhanced efficiency from perceptual processing to motor execution.

In summary, our findings demonstrate robust spatiotemporal associations between EEG microstate dynamics (C/D transitions) and fMRI-based CAP fluctuations within the DAN and VAN. The spatial congruence between microstate D topography and DAN-dominant CAP configurations supports its mechanistic involvement in attention-guided motor reprogramming, consistent with FPN recruitment during precision movement execution [55]. Significant correlations observed between microstate C occurrences and temporal parameters of CAP (DAN/VAN) imply dynamic reconfiguration between the DMN and DAN during rehabilitation-induced neuroplasticity, potentially reflecting a reallocation of resources from self-referential processing to goal-directed motor planning [56]. Considering the intrinsically long-term treatment protocol and substantial medical burden associated with motor rehabilitation for PD [57], the multimodal prognostic framework we developed holds significant translational value as it, through integrating spatiotemporal biomarker prediction models, achieved a high discriminant accuracy (86% accuracy) in stratifying patients’ responsiveness to a 2-week MIRT course, outperforming single-modality predictors and clinical scales (Table 2 and Table S1). It is important to note that our exploratory efforts to establish a cross-cohort EEG microstate template as a universal biomarker for PD rehabilitation revealed inherent limitations in the specificity of the EEG microstate parameters for predicting MIRT outcomes. This may be attributed to the significant individual disease heterogeneity among PD patients and the dual mechanisms of compensation and recovery inherent in the rehabilitation process [16]. Our study, which adheres to the commonly adopted approach of condition-specific EEG microstate templates in PD neuromodulation [23], is better suited to capturing the personalized neuroplasticity trajectories (Table S2). Furthermore, the spatiotemporal features of microstate D and CAP (DAN/VAN) offer quantifiable biomarkers for closed-loop neuromodulation protocols for the adaptive reconstruction of the brain network, facilitating the coordinated application of neurofeedback and repetitive transcranial magnetic stimulation to potentially enhance the efficiency of the rehabilitation process [58,59].

The present study is limited in several ways, but it lays the groundwork for additional future investigations. The primary limitation lies in the absence of a healthy control group as a baseline reference, which may restrict the in-depth analysis of the specific mechanisms underlying short-term motor rehabilitation following MIRT in PD patients. The pharmacological treatment of patients with PD imposes certain limitations on our study findings. Although we endeavored to minimize the potential for dose-related confounding by ensuring a stable medication status and by designing the study to balance medication dosages between the 2 groups to the point of nonsignificance, the established effects of pharmacological interventions on neural activity warrant stricter control in future research. This could include the possibility of conducting pharmacokinetic analyses to accurately assess the relationship between pharmacological interventions and rehabilitation outcomes. Furthermore, examining the impact of medication on EEG and fMRI signals within a controlled environment may reveal the underlying neurophysiological processes. The robustness of the model in different populations needs to be verified by multicenter studies. Moreover, by conducting multi-site trials of noninvasive neuromodulation to further verify the model, we can establish a dose–response relationship between biomarker normalization and clinical symptom relief, which can facilitate clinical translation. Existing biomarkers are inadequate in encapsulating the full neuroplasticity cascade that influences long-term quality of life as measured by the 3-month follow-up using the PDQ-39 (Table S3). Subsequent research is essential in incorporating longitudinal neural activity data, thereby validating the underlying mechanisms that contribute to the enduring efficacy of MIRT treatments.

This study confirms that neuroplasticity driven by attentional network activation and the dynamic reallocation of attentional resources are the core mechanisms by which short-term MIRT facilitates compensatory motor function. This finding underscores the necessity of developing intervention strategies that integrate cognitive–motor dual regulation. By establishing an evaluation framework based on the spatiotemporal dynamics of brain networks, this study provides a generalizable methodological paradigm for monitoring treatment efficacy and exploring mechanisms during the rehabilitation phase of PD and related neurological disorders.

## Data Availability

The data that support the findings of this study are available on request from the corresponding authors but restrictions apply to the availability of these data, which were used under license for the current study, and so are not publicly available.
