# Supplementary material for: Enhancing Attention Network Spatiotemporal Dynamics for Motor Rehabilitation in Parkinson’s Disease
Source: Cyborg Bionic Syst. 2025 Jun 19;6:0293. doi: 10.34133/cbsystems.0293 (PMC12178139; doi:10.34133/cbsystems.0293)
Supplement: Supplementary 1 — Supplementary Methods Figs. S1 to S4 Tables S1 to S3 [file cbsystems.0293.f1.pdf]

Supplementary Material for:

Enhancing Attention Network Spatiotemporal Dynamics for Motor Rehabilitation in Parkinson's Disease

Guangying Pei[1], Mengxuan Hu[1], Jian Ouyang[1], Zhaohui Jin[2], Kexin Wang[1], Detao Meng[2], Yixuan Wang[2], Keke Chen[2], Li Wang[1], Li-Zhi Cao[1*], Shintaro Funahashi[3], Tianyi Yan[1*], Boyan Fang[2*]

This file includes:

Supplementary Methods

Figs. S1 to S3

Tables S1 to S3

Supplementary Methods

Participants

Inclusion criteria: (1) Idiopathic Parkinson's disease diagnosed by a neurologist according to the Movement Disorder Society criteria (Postuma et al., 2015), with Hoehn–Yahr (H-Y) stage ≤ 3; (2) stable vital signs, with no serious cardiopulmonary disease or osteoarthropathy; (3) stable medication, with no drug adjustment within 3 months; (4) if patients had other diseases, they needed no special treatment during hospitalization; (5) no DBS or in vivo implantation treatment; and (6) were able to understand each item of the informed consent, were willing to sign the informed consent, and promised to complete the assessments and treatments.

Exclusion criteria: (1) Patients with fractures or psychotic symptoms; and (2) patients with Clinical Dementia Rating (CDR) (Morris, 1993) scores > 0.5, or with vision or hearing impairments, who were unable to complete the rehabilitation protocol.

MIRT procedure

The 2-week MIRT program was conducted in a hospital setting, 5 days per week, and was composed of four daily rehabilitation sessions. The duration of each session was 30-60 minutes. The MIRT procedure includes four sessions: one-on-one treatment, treadmill training, aerobic training, and speech therapy.1) One-on-one treatment: The 30-minute one-to-one sessions are administered by the hospital's physiotherapist and consist mainly of warm-up activities and active and passive stretch exercises. This section is used to improve the stretch of the abdominal muscles, strengthen the muscles around the spine, adjust posture, and control balance and posture. 2)Treadmill training: Treadmill training was performed using C-MiLL (Motek, Amsterdam/Culemborg, Netherlands) and Balance Tutor (Meditouch, Netanya, Israel) with auditory cues, visual cues, and an anti-interference platform with feedback. This part is 30 minutes in total, performed once in the morning and once in the afternoon, and is used to improve balance and gait. 3)Aerobic training: Aerobic training was performed on upper and lower body trainers (T5XR; Nustep, Ann Arbor, MI, USA) for a total of 30 minutes. 4)Speech therapy: Speech therapy consists of three interventions and lasts for half an hour. Consultation for management of swallowing and language problems; Personal swallowing training instruction for proper handling of food and liquid intake and meal monitoring; Speech therapy for hypokinetic dysarthria focuses on facial exercises to improve mouth movement and facial expression; breathing exercises to reduce speech stress; and exercises to improve vocalization, articulation, and rhythm of speech.

MRI Data Preprocessing

After the first ten time points were discarded, blood oxygen level-dependent functional images were corrected for timing, realigned, registered to the corresponding T1-weighted images, normalized to the Montreal Neurological Institute template, resampled to 3 mm×3 mm×3 mm, processed to remove nuisance covariates, and smoothed with a 4-mm full width at half maximum Gaussian kernel. Specifically, the

global mean signal, white matter signal, cerebrospinal fluid signal, and global Friston 12-motion parameters (6 motion parameters and 6 motion derivatives) were regressed out as nuisance covariates. Structural T1-weighted images were obtained using a three-dimensional brain volume sequence (echo time = 3.06 ms, repetition time = 8.06 ms, inversion time = 450 ms, flip angle = 15°, field of view = 300×300 mm2, matrix = 512×512, slice thickness = 1.0 mm, and slice number = 160).

Dynamic Brain States and Metrics by Coactivation Patterns Analysis

The variance gain method was used to determine the optimal number of clusters, while the test-retest reliability method was used to evaluate the stability of the K value in this study. **Fig. S1(A)** plots the variance explained by clusters (between-cluster variance divided by the sum of between-cluster variance and within-cluster variance) for k=2-16. When k increases from k-1 to k, the variance gain explained by k-means clustering gradually decreases, and the explained variance gain levels off when k>12. Furthermore, the test-retest reliability method was used to further evaluate the cluster number's stability, and the average centroid similarity was obtained after 100 repetitions. The results are shown in **Fig. S1(B)**. As the K value increases, the similarity decreases. K =6, 8, 10, 13, 14, 15, 16 are local maximum values.

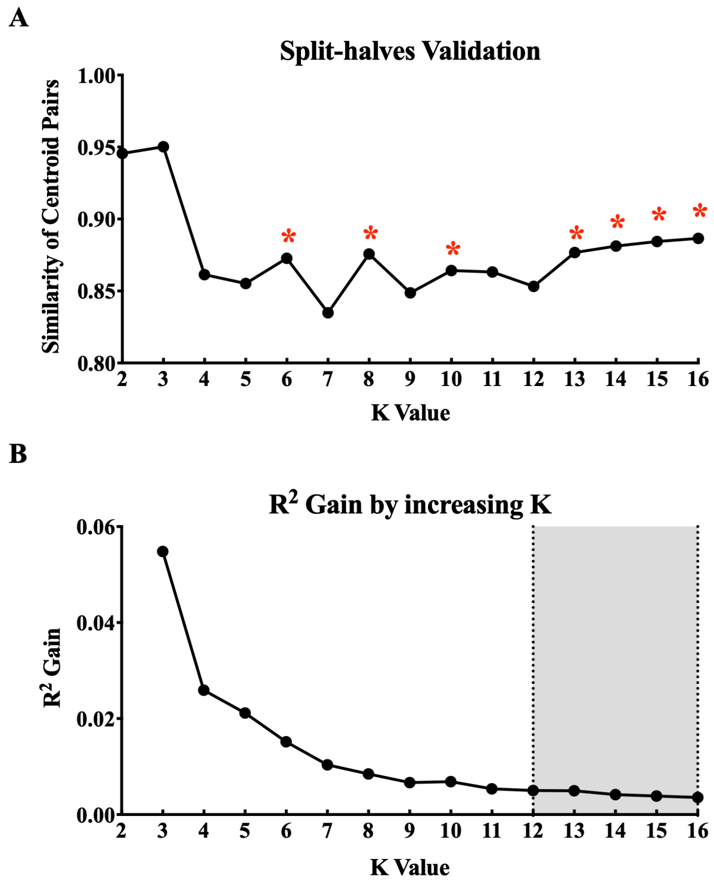

Fig. S1. Stability evaluation of K value. Results of the variance explained (A) and test-retest reliability (B) for different K values.

Supplementary Results

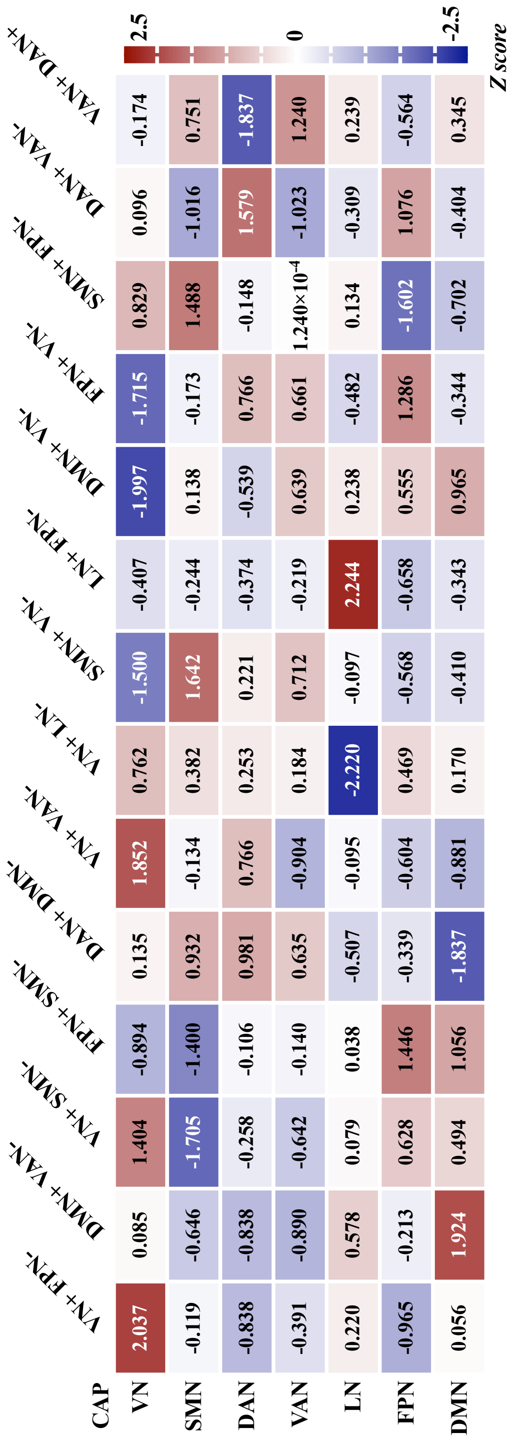

Fig. S2. The z scores of each kind of network of group CAPs. The network of each CAP with the largest z score is defined as the coactivated network, and the network with the smallest z score is defined as the deactivated network. "+" means "coactivation", and "-" means "deactivation". VN: visual network; SMN: somatomotor network; DAN: dorsal attention network; VAN: ventral attention network; LN: limbic network; FPN: frontoparietal network; DMN: default-mode network; CAP: coactivation pattern.

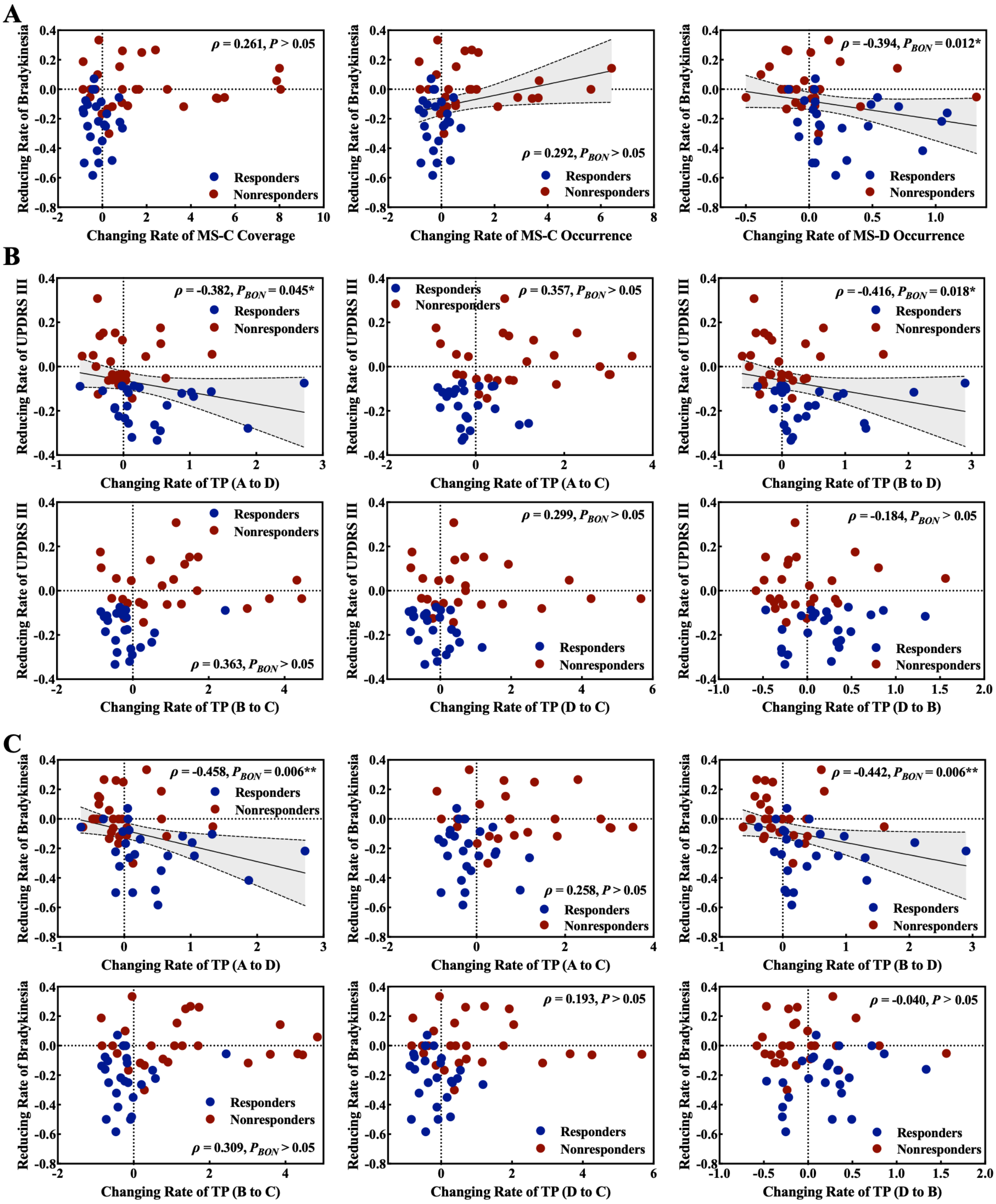

Fig. S3. EEG microstate clinical correlations. (A) Scatter plots display the correlation between the bradykinesia subscale and EEG microstate parameters with significant differences. **(B)** Scatter plots display the correlation between the MDS-UPDRS III and EEG transition probabilities with significant differences. **(C)** Scatter plots display the correlation between the bradykinesia subscale and EEG transition probabilities with significant differences. *, corrected P < 0.05; **, corrected P < 0.05.

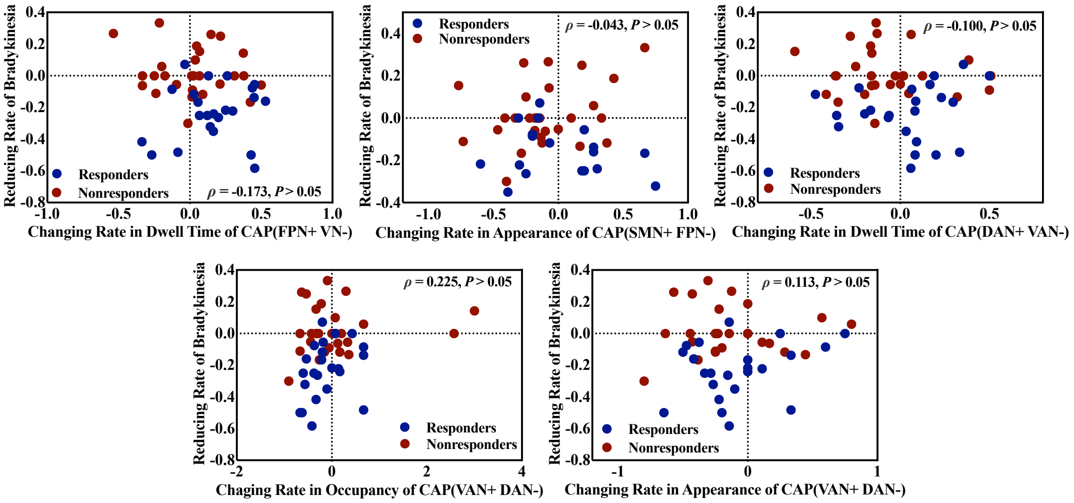

Fig. S4. The fMRI CAP temporal parameters Bradykinesia subscale correlations. Scatter plots display the correlation between the bradykinesia subscale and fMRI CAP temporal parameters microstate parameters with significant differences.

Table S1. Neuroplastic outcomes from pre- to post-intervention in Parkinson's disease

Reference	Intervention	Measure neuroplasticity	Specification signal clinical corrolation
Lamoš M, et al.[1]	DBS	EEG microstate	EEG microstate D GEV vs UPDRS III ($r = 0.400$, $p = 0.040$)
Liu S, et al.[2]	rTMS	EEG microstate	EEG microstate D coverage vs UPDRS III ($r = -0.497$, $p = 0.011$)
Liu T, et al.[3]	tACS	fMRI CAP	FPN activation values vs UPDRS-III ($r = −0.439$, $p = 0.039$)
Li T, et al.[4]	MIRT	fMRI: BOLD signal, ASL	CBF/fALFF ratio in the right superior grontal gyrus (dorsolateral) vs UPDRS III ($r = −0.389$, $p = 0.020$)
Evangelisti, et al.[5]	Dopamine	EEG-fMRI	SMN, DMN and DAN enhanced connectivity, no clinical correlation analysis
Our work	MIRT	EEG microstate and fMRI CAP	EEG microstate C occurrence vs UPDRS III ($r = 0.409$, $p = 0.027$); EEG microstate D occurrence vs UPDRS III ($r = -0.393$, $p = 0.036$); CAP(DAN+ VAN-) dwell time vs UPDRS III ($r = -0.334$, $p = 0.016$); Multisignal prediction MIRT efficacy (accuracy: 86%)

Referrences

[1]Lamoš M, Bočková M, Goldemundová S, et al. The effect of deep brain stimulation in Parkinson’s disease reflected in EEG microstates[J]. npj Parkinson's Disease, 2023, 9(1): 63.

[2]Liu S, Yang S, Feng K, et al. A Study on the effects of repetitive transcranial magnetic stimulation on EEG microstate in patients with Parkinson’s disease[J]. IEEE Transactions on Neural Systems and Rehabilitation Engineering, 2024.

[3]Liu T, Yan Z, Han Z, et al. Cortico–subcortical spatiotemporal dynamics in Parkinson’s disease can be modulated by transcranial alternating current stimulation[J]. Brain Science Advances, 2023, 9(2): 114-135.

[4]Li T, Wang L, Piao Z, et al. Altered neurovascular coupling for multidisciplinary intensive rehabilitation in Parkinson's disease[J]. Journal of Neuroscience, 2023, 43(7): 1256-1266.

[5]Evangelisti S, Pittau F, Testa C, et al. L-dopa modulation of brain connectivity in Parkinson’s disease patients: A pilot EEG-fMRI study[J]. Frontiers in neuroscience, 2019, 13: 611.

Table S2. Accuracy, precision, and sensitivity of the SVM classifier based on 5 times 5-fold cross-validation (mean±SD)

EEG microstate parameters	Accuracy (%)	Precision (%)	Sensitivity (%)
**Acorss-group (responders and nonresponders)[a]**	78.00 ± 2.00	78.53 ± 4.21	85.83 ± 4.08
**Our work (specific responders or nonresponders)[b]**	82.66 ± 3.23	85.29 ± 6.16	78.45 ± 2.45

Note：

a: A repeated - measures analysis of variance was performed, with group (responders vs. non-responders) as the between-subjects factor and time point (pre- vs. post- MIRT) as the within - subjects factor. There was no significant interaction effect of MIRT × group for the microstate parameters ($F = 0.630$, $P = 0.706$, $\eta_p^2 = 0.077$); Parameters (coverage, duration, occurrence) of all EEG microstates (class A, B, C, and D) were selected and input into support vector machine. 5 repetitions of 5-fold cross-validation were used to assess performance, and mean accuracy, precision, and sensitivity are reported.

b: EEG microstate C occurrence and D occurrence, which were significantly correlated with MDS-UPDRS III, were selected and input into support vector machine. 5 repetitions of 5-fold cross-validation were used to assess performance, and mean accuracy, precision, and sensitivity are reported.

Table S3. Spatiotemporal Brain Metrics PDQ-39 Correlations

	Spatiotemporal Brain Metrics	Spearman Correlations
EEG	Microstate C coverage	$\rho = 0.011, P > 0.05$
	Microstate C occurrence	$\rho = 0.045, P > 0.05$
	Microstate D occurrence	$\rho = 0.006, P > 0.05$
	Microstate A → D transition probability	$\rho = -0.047, P > 0.05$
	Microstate A → C transition probability	$\rho = 0.060, P > 0.05$
	Microstate B → D transition probability	$\rho = 0.043, P > 0.05$
	Microstate B → C transition probability	$\rho = -0.059, P > 0.05$
	Microstate D → C transition probability	$\rho = 0.003, P > 0.05$
	Microstate D → B transition probability	$\rho = 0.031, P > 0.05$
fMRI	CAP(FPN+ VN-) Dwell time	$\rho = -0.299, P_{BON} > 0.05$
	CAP(SMN+ FPN-) Appearance	$\rho = -0.042, P > 0.05$
	CAP(DAN+ VAN-) Dwell time	$\rho = -0.029, P > 0.05$
	CAP(VAN+ DAN-) Occupancy	$\rho = 0.169, P > 0.05$
	CAP(VAN+ DAN-) Appearance	$\rho = 0.133, P > 0.05$
